# Relationship Between Socio-Demographic Characteristics, Reproductive Health Behaviors, and Health Literacy of Women in Serbia

**DOI:** 10.3389/fpubh.2021.629051

**Published:** 2021-04-29

**Authors:** Milena Maricic, Goran Stojanovic, Vanja Pazun, Milos Stepović, Ognjen Djordjevic, Ivana Zivanovic Macuzic, Vesna Milicic, Veroljub Vucic, Svetlana Radevic, Snezana Radovanovic

**Affiliations:** ^1^Department School of Applied Health Science Studies, Academy of Applied Studies Belgrade, Belgrade, Serbia; ^2^Faculty of Medical Sciences, University of Kragujevac, Kragujevac, Serbia; ^3^Department of Anatomy, Faculty of Medical Sciences, University of Kragujevac, Kragujevac, Serbia; ^4^Department of Dermatovenerology, Faculty of Medical Sciences, University of Kragujevac, Kragujevac, Serbia; ^5^Health Center Trstenik, Trstenik, Serbia; ^6^Department of Social Medicine, Faculty of Medical Sciences, University of Kragujevac, Kragujevac, Serbia

**Keywords:** health literacy, reproductive health, behavior, women, Serbia

## Abstract

**Introduction:** Health literacy of women can significantly affect different aspects of reproductive health. The aim of this study was the assessment of relationship of health literacy, socio-demographic characteristics and reproductive health behaviors of women in Serbia.

**Methodology:** This was a cross-sectional study on a random sample of women aged 18 and over from the territories of three Serbian regions, stratified according to age groups, region and type of settlement. A standardized version of the European Health Literacy Survey Questionnaire (HLS-EU-Q47) was used in assessing health literacy of women.

**Results:** Based on the calculated index of health literacy, 9.6% of respondents had inadequate health literacy. Inadequate levels of health literacy were more common in women living in rural areas (OR = 1.111) and the poorer classes (OR = 5.122). Employed women (OP = 1.249), with good health (OR = 1.512) with a degree (OR = 1.535) had bigger odds to have adequate health literacy. Multivariate regression analysis showed the following significant predictors: commitment to the chosen gynecologist (OR = 1.530), contraceptive use (OR = 1.020), knowledge of the damages that could be caused by the human papillomavirus (HPV) (OR = 1.578), awareness of vaccine availability against HPV infection (OR = 1.217) and following the health-related topics (OR = 2.350).

**Conclusion:** Limited levels of health literacy were significantly higher among middle-aged women, among those living in rural areas, among women who rated their health as poor or very poor, and who exhibited more negative patterns of reproductive health behavior, indicating the need for implementation of prevention programs and strategies with the aim of increasing the level of health literacy.

## Introduction

The World Health Organization defines health literacy as the cognitive and social skills and capacities needed to gain access to, understand and use information in ways which promote and protect good health (1, 2). This is a term of increasing importance in the field of public health and health care. It implies placing our own health, health of the family and of the community in the context of understanding the factors that affect health, as well as the knowledge about how to deal with the preservation and improvement of health (3, 4). Numerous studies showed that low levels of health literacy caused more frequent use of the health service and administration of drugs (5), poorer self-assessment of health and ability to interpret medical posts, poorer ability to manage chronic diseases (6, 7), less chance to participate in preventive activities (8), poorer health outcomes (9), and poorer communication with health workers (10).

The results of European health literacy surveys showed significant differences between countries, indicating that some countries not only had lower health literacy, but also greater inequality in terms of the distribution of health literacy within their population with the prevalence of limited health literacy which varies from 28.7% in the Netherlands to 44.8% in Greece, 56.4% in Austria, 46.3% in Germany, 40% in Ireland, 44.6% in Poland, 58.3% in Spain, 62.1% in Bulgaria (3). Inadequate health literacy were present in 17.4% respondents in the Republic of Srpska, Bosnia and Herzegovina and one-fifth of the participants in Albania, transitional country in the Western Balkans (11, 12).

The fact that health literacy affected the quality of life of patients was also shown by the results of research conducted in our country, where health literacy was identified as a very important predictor of quality of life. The data also showed that more than half of the respondents (64%) had limited health literacy. The lowest mean health literacy index (28.01 ± 9.34) was within the disease prevention dimension, where the largest number of respondents showed limited health literacy (70%) (13, 14).

As regards a better health literacy score, it was found among the following participants: younger, employed, and those with a high level of education, a good self-perception of health, a good socioeconomic status and no chronic conditions (15, 16). Other health literacy studies showed that inadequate or marginal health literacy was present in (44.1%) participants and adequate health literacy was present in (55.9%) participants (17).

In our country a lot of things are being done in order to improve reproductive health behaviors in the female population. Relevant national authorities and institutions are undertaking numerous activities through various national programs and strategies for the purpose of further enhancing the preventive measures and protection of women's reproductive health, such as the National Strategy for the Fight against human immunodeficiency virus infection and acquired immunodeficiency syndrome (HIV/AIDS) HIV/AIDS, the purpose of which is not only related to monitoring HIV infected persons and HIV or AIDS related deaths, but it is also related to voluntary confidential counseling and HIV testing (18). In addition, the Ministry of Health of the Republic of Serbia and the network of Institutes of Public Health in Serbia use various national breast and cervical cancer early detection programs and strategies for the purpose of continually undertaking preventive measures among the population of women (19). Also, in the period 1997–2007 at about 60% of primary health care centers, the following recommendations of the “Program of Promotion, Support and Care for Breastfeeding and BabyFriendly and Mother-Friendly Health Institutions” “Schools for Pregnant Women and Parenting” were set up, and in about 30% of primary health centers services such as psycho-physical preparation for pregnancy and delivery were provided as well (20). Furthermore, providers of reproductive health services at all levels of healthcare protection in our country—actively participate in raising the level of awareness of health culture and women's reproductive health knowledge by means of different education programs regarding family planning, sexually transmitted disease (STD) prevention, women's health protection during pregnancy and post-partum period. Their play a highly significant role, but the results of a considerable number of women's health studies conducted in our country indicate that the risk level of women's health is still high (21).

According to the data obtained by the National Health Survey of the Population of Serbia, 62.8% of women aged 15 and over had their own gynecologist, 56.2% of women were sexually active, whereas only 14.8% used a condom during the last sexual intercourse. Women of reproductive age from suburban settlements (39.2%), married (45.0%), and employed (39.1%) use insecure methods of contraception significantly more often. Highly educated respondents, employed, of good financial status and from urban areas visited gynecologists more often and most often performed screening examinations on their own initiative (22). The knowledge about contraception methods among girls living in the Roma settlements lags behind the average for overall population of women (15–49 years), which indicates low transfer of knowledge from older to younger generations (23).

According to the Serbia Multiple Indicator Cluster Survey—MICS, the reported rate of usage of modern contraceptive methods is 22%. The most popular method among modern contraceptives methods is a condom, whereas only 3–4% of female respondents use intrauterine devices (IUD), mirena or hormonal contraception. Also, 7% of married women have not satisfied the need for modern contraception. Each reported live birth is followed by five induced abortions (24).

Therefore, it is necessary to encourage further research in the field of reproductive health, for the purpose of getting a better insight into the measures that are supposed to be undertaken. Accordingly, the purpose of our study was to assess the association between health literacy of women and sociodemographic characteristics, behaviors and reproductive health knowledge of the female population in Serbia—in accordance with the recommendations of the European Commission related to the necessity to conduct an increasing number of studies all over Europe for the purpose of adequate standardization of comparisons and summaries of health literacy results among different groups of respondents.

In our country, there are no health literacy results that refer strictly to the population of women, which contributes to the topicality of the results obtained by this research. Additionally, the potential impact of health literacy on women's reproductive health behavior has not been investigated in our country yet. The aim of this study was the assessment of relationship of health literacy, socio-demographic characteristics and reproductive health behaviors of women in Serbia.

## Materials and Methods

### Type of Study and Population

The research was conducted as a cross-sectional study. The population included women aged 18 years and older. The respondents were introduced with the research objectives and procedures. The respondents filled questionnaires anonymously. After the respondents signed informed consent, an evaluation of eligibility for inclusion in the study was conducted. Suitability assessment was based on the inclusion and exclusion criteria. The respondents had to meet all the inclusion criteria and none of the exclusion criteria in order to participate in the study.

The inclusion criteria in the study were the following: signed informed consent, female respondents aged 18 years or more who were capable of understanding the nature of the study. The non-inclusion criteria were the following: women younger than 18 years who were incapable of understanding the study protocol.

This research was conducted through the Junior Project (Number 09/18) of the Faculty of Medical Sciences. The research protocols used in this research were approved by the Ethics Committee of the Faculty of Medical Sciences, University of Kragujevac.

### Sampling

This was a cross-sectional study on a random sample of women aged 18 and over from the territory of three regions (Vojvodina, Belgrade and Šumadija district), stratified according to age groups, region and place of residence. By including different regions and places of residence, we wanted to examine whether there were significant differences in health literacy that could certainly be conditioned by the place of residence, given that the population coming from different environments show differences in habits, attitudes, customs and culture. Villages were marked as rural areas. For the purpose of testing the health literacy of women, a standardized version of the European Health Literacy Survey Questionnaire (HLS-EU-Q47) was used, which is publicly available and is free to use as long as specific recommendations are followed (3). The questionnaire has already been used to test the health literacy of certain population groups in our region (14, 17).

The HLS-EU-Q-47 consisted of 47 items dedicated to accessibility, understanding, evaluation, and handling information in relation to health across three subdomains: disease prevention, health protection, and health promotion. The health literacy of women was measured by the Likert scale. For each item, respondents rated the perceived difficulty of a given task or situation on a four-category Likert scale (1 = very difficult, 2 = difficult, 3 = easy, 4 = very easy), with the lowest possible average score of 1 and the highest possible mean score of 4. For the respondents who gave valid answers to at least 80% of all questions, general health literacy index was calculated (HLS-EU Index-Q) according to the formula Index = [mean (per item – 1) × (50/3)], the values of which ranged from a minimum of 0 to a maximum of 50, where 0 represented “the lowest possible” and 50 represented the “best possible” health literacy score. Based on index thresholds, respondents were divided according to the four health literacy levels: “inadequate” (0–25), “problematic” (>25–33), “sufficient” (>33–42), and “excellent” (>42–50) health literacy.

Socio-economic characteristics of the respondents and the characteristics of reproductive health behaviors were assessed using an additional questionnaire created for the purposes of this research. The additional questionnaire contained questions related to demographic characteristics (age, marital status, education, type of settlement), socio-economic characteristics (employment status, self-assessment of financial status), reproductive health behaviors of respondents [contraceptive use, participation in the Papanicolaou-test (PAP-test), participation in the mammogram, knowledge about HIV and about HIV testing, the knowledge of possible manifestations that can be caused by the human papillomavirus (HPV), the knowledge of the existence of a vaccine against HPV and against the hepatitis B], questions related to the use of women's health care services and to the self-assessment of health. The response rate was 97%.

The independent variables in the study were demographic and socioeconomic characteristics of respondents (age, family structure, type, region, level of education, employment, household economic status, health self-assessment) and characteristics of reproductive health behavior (use of contraceptives, participation in the PAP-test, participation in the mammogram, knowledge about HIV and HIV testing, the knowledge of possible manifestations that can be caused by HPV, awareness of vaccine availability against HPV infection and against the hepatitis B), while the dependent variable was health literacy. For the purpose of univariate and multivariate logistic regression analysis, respondents were classified into two categories of health literacy: the “inadequate” and “problematic” levels were combined to a single level, which we referred to as “limited health literacy” (0–33) whereas, “sufficient” (>33–42) and “excellent” (>42–50) levels were combined to a single level, called “adequate health literacy” (34–50).

### Statistical Analysis

Statistical analysis of results was presented and analyzed in the appropriate mathematical-statistical methods according to the type of data. The difference in the prevalence of categorical variables was tested using the Chi-Square Tests. The connection between characteristics of reproductive health behavior of respondents as independent variables, and health literacy as dependent variable, was tested by bivariate and multivariate logistic regression. A value of *p* < 0.05 was considered statistically significant. Statistical analysis was performed by using commercial standard SPSS software package Version 19.0. (The Statistical Package for the Social Sciences software) (Version 19.0., SPSS Inc., Chicago, IL).

## Results

A total of 776 female subjects aged 18 years and older were surveyed in this study. In terms of education level, 56.7% of women completed secondary education. The sample included 49.3% married women, 69.5% employed and 56.8% of women with medium material status. Over half of them came from urban areas (52.9%). Based on the calculated index of health literacy, 9.6% of respondents had inadequate health literacy, 14.5% had problematic, 37.2% had sufficient, and 38.7% had excellent health literacy.

Differences in health literacy levels by demographic and socioeconomic characteristics of the women were shown in Table 1. There were significant differences in health literacy levels among women of different age, as well as by type of settlement and health self-assessment. Middle-aged women (35–54 years), women from rural areas (57.7%), and those who rated their health as poor (31%) and very poor (36.5%) had significantly higher levels of inadequate health literacy. Viewed by region, there was no significant difference in health literacy levels (Table 1).

**Table 1:** Sociodemographic characteristics of women in Serbia by health literacy levels.

**Variables**	**Women (%)**	**Health literacy**				p^*^
		**Inadequate**	**Problematic**	**Sufficient**	**Excellent**	
**Age (years)**						
<25	17.1	4.2	20.0	17.3	18.7	0.001
25–34	17.5	8.5	12.6	18.8	20.7	
35–44	24.1	32.4	27.4	23.2	21.4	
45–54	24.4	31.0	26.7	23.6	22.5	
55–64	13.1	18.3	8.1	13.3	14.0	
65+	3.7	5.6	5.2	3.8	2.7	
**Regions**						
Vojvodina	39.4	30.8	29.8	35.6	34.4	0.552
Belgrade	39.4	36.0	35.0	32.9	31.3	
Sumadija district	21.2	33.2	36.2	31.5	34.3	
**Education**						
Elementary school or lower	6.6	5.6	8.9	7.0	5.4	0.786
Middle school	56.7	60.6	53.3	55.0	58.9	
High school/college or higher	36.7	33.8	37.8	38.0	35.7	
**Marital status**						
Never married	37.9	38.6	37.4	38.6	37.4	0.189
Married	49.3	42.8	48.9	48.5	51.9	
Separated/divorced	10.7	12.9	12.2	11.0	9.3	
Widowed	2.0	5.7	1.5	1.9	1.4	
**Employment status**						
Employed	69.5	69.8	67.4	70.6	9.5	0.920
Unemployed	30.5	31.2	32.6	30.0	30.5	
**Financial status perception**						
Very good	1.4	4.2	2.2	1.1	0.7	0.001
Good	6.7	18.3	6.7	5.9	4.7	
Average	56.8	46.5	60.0	56.5	58.1	
Bad	28.0	26.8	26.7	31.7	25.4	
Very bad	7.1	4.2	4.4	4.8	11.1	
**Place of residence**						
Rural	52.9	57.7	51.1	50.9	54.4	0.001
Urban	47.1	42.3	48.9	49.1	45.6	
**Health self-assessment**						
Very good	13.7	9.9	14.4	11.1	17.8	0.001
Good	32.8	2.8	6.6	48.3	43.6	
Average	37.4	9.8	10.5	35.1	33.9	
Bad	5.8	31.0	38.5	4.5	2.7	
Very bad	1.3	36.5	32.0	1.0	2.0	

* *Chi-Square tests*.

Results of univariate regression analysis related to demographic and socio-economic characteristics of female respondents showed that the most important predictors of health literacy levels were: the type of settlement, education, financial status, employment status and health status. Women with the highest level of education had a 43% higher possibility to have higher health literacy level compared to women with the lowest levels of education (OR = 1.435). Inadequate levels of health literacy were 11% more common in women living in rural areas (OR = 1.111). The proportion of women who had inadequate health literacy was inversely proportional to the degree of their education. In fact, women with a degree were 1.5 times more likely to have adequate health literacy as compared to those with lower educational status (OR = 1.535). With regard to the index of well-being, the poorer classes were 5 times more likely to have inadequate health literacy in comparison to those belonging to the richest classes (OR = 5.122). Employed women had 12% bigger odds to have adequate health literacy if compared to the unemployed ones (OP = 1.249). Women with good health were 15% more likely to be in the group of women with adequate health literacy compared to the women with bad health status (OR = 1.512). Multivariate regression analyses showed the following significant predictors of health literacy: education, financial situation, and health status (Table 2).

**Table 2:** Cross odds ratio (OR) and 95% confidence intervals (CI) for the health literacy levels with respect to demographic and socioeconomic characteristics of women in Serbia.

**Variables**	**Univariate model**		**Multivariate model**	
	**OR (95% CI)**	*P*-value^*^	**OR (95% CI)**	*P-*value^*^
**Age (years)**				
<25	0.442 (0.144–1.326)	0.126	0.571 (0.154–2.055)	0.493
25–34	0.336 (0.128–0.863)	0.032	0.412 (0.116–1.44)	0.152
35–44	1.118 (0.415–2.891)	0.809	1.257 (0.345–4.042)	0.612
45–54	0.663 (0.232–1.801)	0.343	0.638 (0.211–2.361)	0.607
55–64	0.566 (0.187–1.663)	0.321	0.613 (0.162–1.982)	0.405
65+	1		1	
**Marital status**				
Never married	0.643 (0.257–1.512)	0.327	0.858 (0.343–2.118)	0.624
Married/common-law marriage	0.542 (0.246–1.231)	0.146	0.544 (0.284–1.126)	0.352
Separated/divorced	0.747 (0.314–2.217)	0.712	0.812 (0.282–2.210)	0.713
Widowed	1		1	
**Education**				
Elementary school or lower	1.535 (0.656–3.187)	<0.001	1.282 (0.532–3.143)	<0.001
Middle school	1.173 (0.721–1.531)	<0.001	1.215 (0.514–1.526)	<0.001
High school/college or higher	1		1	
**Place of residence**				
Rural	1.111 (0.664–1.713)	<0.001	1.173 (0.634–1.506)	<0.001
Urban	1		1	
**Employment status**				
Unemployed	1.249 (0.662–1.813)	<0.001	1.213 (0.732–2.154)	<0.001
Employed	1		1	
**Financial status perception**				
Very good	5.122 (0.867–18.448)	<0.001	2.62 (0.402–15.323)	<0.001
Good	3.142 (0.969–9.657)	<0.001	2.12 (0.627–7.344)	<0.001
Average	1.276 (0.5012–3.297)	<0.001	1.328 (0.38–2.628)	<0.001
Bad	1.059 (0.402–2.806)	<0.001	1.05 (0.365–2.278)	<0.001
Very bad	1		1	
**Health self-assessment**				
Very good	1.512 (1.302–1.611)	<0.001	1.266 (1.141–1.411)	<0.001
Good	1.133 (0.359–3.453)	<0.001	0.752 (0.549–1.110)	<0.001
Average	1.364 (0.659–2.842)	<0.001	1.121 (1.033–1.390)	<0.001
Bad	0.751 (0.434–1.813)	<0.001	1.231 (1.006–1.434)	<0.001
Very bad	1		1	

* *Logistic regressions—the reference category is adequate health literacy*.

In relation to the surveyed characteristics of reproductive health behaviors and enlightenment, significant differences in health literacy levels were observed among women who did not use any method of protection against sexually transmitted diseases and unintended pregnancies, as well as among those who did not know where to get tested for HIV, or in those who did not know what damage could be caused by the HPV, as well as in those who were not aware of HPV or hepatitis B vaccine availability (Table 3).

**Table 3:** Reproductive health characteristics, health behavior, and levels of health literacy among women in Serbia.

**Variables**	**Women**	**Inadequate**	**Problematic**	**Sufficient**	**Excellent**	p^*^
**Do you have your chosen gynecologist?**						
Yes	86.2	90.1	85.2	85.6	86.2	0.771
No	13.8	9.9	14.8	14.4	13.8	
**Do you use contraceptives?**						
Yes	39.2	32.1	49.1	52.8	61.2	0.001
No	60.8	67.9	50.9	47.2	38.8	
**Have you ever gone on a mammogram?**						
Yes	31.6	36.6	32.8	29.2	32	0.804
No	68.4	63.4	67.2	70.8	68	
**Have you gone on a mammogram?**						
Voluntarily	24.8	28.3	27.4	22.7	24.5	0.989
Organized screening	21	19.6	20.2	24	19.1	
By the doctor's invitation	6.8	6.5	6	7.1	6.9	
Did not know	47.3	45.6	46.4	46.8	49.5	
**Have you ever undergone the PAP-test?**						
Yes	81.2	80.7	82.9	84	78.5	0.572
No	18.8	19.3	17.1	16	21.5	
**Have you undergone the PAP-test?**						
Voluntarily	46.3	49.3	50.7	48.7	52.6	0.313
Organized screening	29.7	24.6	28.2	29.7	31.6	
By the doctor's invitation	9	8.7	8.1	8.9	11	
Did not know	15.1	17.4	10	12.7	4.7	
**Do you know where you can be tested for HIV?**						
Yes	61.9	49.3	39.3	67.5	60.4	0.001
No	38.1	50.7	60.7	32.5	39.6	
**Have you ever been tested for HIV?**						
Yes	12.9	12.1	8.9	15.5	13	0.303
No	87.1	87.9	91.1	84.5	87	
**Do you know what may cause the HPV?**						
Yes	64.6	52.2	60.7	64.5	69.3	0.001
No	35.4	47.8	39.3	35.5	30.7	
**Is there a vaccine against HPV?**						
Yes	42.1	33.8	38.5	42.8	45.2	0.001
No	57.9	64.2	61.5	57.2	54.8	
**Is there a vaccine against hepatitis B?**						
Yes	57.5	52.1	53.3	60.4	62.4	0.001
No	42.5	47.9	46.7	39.6	37.6	
**Do you follow health-related topics?**						
Yes	45.7	36.6	37.8	38.4	47.8	0.001
No	54.3	63.4	62.2	61.6	42.2	

* *Chi-Square tests*.

*PAP, Papanicolaou-test; HPV, human papilloma virus; HIV, Human immunodeficiency virus*.

Results of the univariate regression analysis showed that significant predictors of health literacy levels were associated with the contraceptive use, possible participation in the PAP-test, knowledge about HIV and HIV testing, the knowledge of possible manifestations that could be caused by the HPV, the awareness of vaccine availability against HPV infection and against the hepatitis B. Women with the appropriate health literacy were 1.9 times more likely to use contraceptives (OR = 1.920) and 1.7 times more likely to be subjected to the PAP-test, than those with a lower health literacy level (OR = 1.705). The subjects with inadequate health literacy were 1.8 times more likely to undergo the PAP-test after being called by their physician than to do it by self-initiative (OR = 1.889). Women with adequate literacy were 1.7 times more likely to know where to test themselves for HIV (OR = 1.706). They were also 1.6 times more likely to know what the human papillomavirus caused (OR = 1.667) and were 1.7 times more likely to know about the HPV vaccine (OR = 1.743) compared to women with the insufficient health literacy level. Women who were not aware of vaccine availability against hepatitis B were 76% more likely to belong to group of those who had inadequate health literacy (OR = 1.763). Women with adequate health literacy were 2.2 times more likely to follow the health-related themes compared to the women with inadequate health literacy, while the multivariate regression analysis showed the following significant predictors: commitment to the chosen gynecologist (OR = 1.530), contraceptive use (OR = 1.020), knowledge of the damages that could be caused by the HPV (OR = 1.578), awareness of vaccine availability against HPV infection (OR = 1.217) and following the health-related topics (OR = 2.350) (Table 4).

**Table 4:** Cross odds ratio (OR) and 95% confidence intervals (CI) for the health literacy levels with respect to reproductive health behaviors of women in Serbia.

**Variables**	**Univariate model**		**Multivariate model**	
	**OR (95% CI)**	*P*-value^*^	**OR (95% CI)**	*P-*value^*^
**Do you have your chosen gynecologist?**				
Yes	1		1	
No	0.185 (0.679–1.733)	0.743	1.530 (0.733–3.212)	<0.001
**Do you use contraceptives?**				
Yes	1		1	
No	1.920 (1.62–2.26)	<0.001	1.02 (0.75–1.39)	<0.001
**Have you ever gone on a mammogram?**				
Yes	1		1	
No	0.796 (0.203–3.122)	0.542	0.724 (0.125–4.201)	0.719
**Have you gone on a mammogram?**				
Voluntarily	1		1	
Organized screening	0.215 (0.743–1.986)	0.534	0.597 (0.187–1.908)	
By the doctor's invitation	0.974 (0.569–1.665)	0.521	0.708 (0.017–2.960)	0.223
**Have you ever undergone the PAP-test?**				
Yes	1		1	
No	1.705 (0.564–5.157)	<0.001	0.410 (0.099–1.693)	0.218
**Have you undergone a PAP-test?**				
Voluntarily	1		1	
Organized screening	1.178 (0.731–1.899)	<0.001	0.803 (0.356–1.810)	0.612
By invitation of doctor	1.889 (0.529–1.494)	<0.001	0.611 (0.191–1.954)	0.472
**Do you know where you can be tested for HIV?**				
Yes	1		1	
No	1.746 (0.539–1.032)	<0.001	0.792 (0.479–1.312)	0.366
**Have you ever been tested for HIV?**				
Yes	1		1	0.252
No	0.706 (0.424–1.175)	0.321	0.640 (0.299–1.372)	
**Do you know what may cause the human papilloma virus (HPV)?**				
Yes	1		1	
No	1.667 (0.384–1.158)	<0.001	1.578 (0.325–1.030)	<0.001
**Is there a vaccine against HPV?**				
Yes	1		1	
No	1.743 (0.531–1.038)	<0.001	1.217 (0.570–2.597)	<0.001
**Is there a vaccine against hepatitis B?**				
Yes	1		1	
No	1.763 (0.549–1.061)	<0.001	0.871 (0.532–1.425)	0.955
**Do you follow health-related topics?**				
Yes	1		1	
No	2.26 (1.83–2.79)	<0.001	2.35 (1.88–2.93)	<0.001

* *The reference category is: adequate*.

## Discussion

Women's health is the result of complex interaction of genetic, biological, physiological, medical, and social factors (25, 26).

In addition to women's general health, reproductive health is also very important. Women's reproductive health is a complex concept that encompasses many aspects of good health: well-being in the area of sexual relations, family planning, protection against adverse events, resources for its preservation, and promotion. Identifying the factors that determine women's reproductive health and behaviors is very important from a health policy perspective (27–29).

Undoubtedly, the concept of health literacy is also closely associated with the knowledge about reproductive health and can negatively affect different aspects of women's reproductive health. Health literacy can significantly determine women's reproductive health behaviors. Several studies demonstrated that women with lower levels of health literacy showed negative health behaviors related to contraceptive use (risks and proper time and way of contraceptive use), screening, initiation of prenatal care, breastfeeding, smoking during pregnancy and postnatal period, and use of health care services in the area of reproductive health (30–33).

Likewise, other studies revealed that women with low health literacy were associated with decreased knowledge of the meaning, mechanisms of action and risks of oral contraception, they had decreased understanding of indications for, timing of, and contraindications of taking emergency contraception, and had four times more chances of not knowing when a woman could get pregnant during her menstrual cycle and that accordingly, they were at higher risk for unplanned pregnancy (34).

Studies examining the relationship between health literacy, sexual behaviors and sexually transmitted diseases reached the conclusion that women with lower health literacy were associated with earlier sexual debut, they more frequently reported unprotected intercourse during the first sexual intercourse (or sexual debut) and were more likely to report multiple sex partners (two or more), compared to those who had higher health literacy (9).

Those results are in line with our research where women with a low level of health literacy did not use contraceptives properly and had more unprotected sexual activities.

Women with low health literacy were associated with increased perception of risk and negative beliefs toward the use of medications and supplements during pregnancy, which resulted in the increased rates of non-adherence to prescribed medications during pregnancy (35), but they expressed increased desire to seek more information on the use of medications during pregnancy, compared to women with adequate health literacy (36). Other studies showed that pharmacists could give a significant contribution in recommendations for treatment of mild health problems in pregnancy, as well as in prevention of unsafe drug use trough the consultation with proscribers or by recommendation of safe over-the-counter OTC drugs (37).

Women with inadequate health literacy had greater smoking rates during pregnancy, lower breastfeeding rates, were more likely to suffer from post-partum depression, whereas women with high health literacy were associated with increased knowledge and concern about the health effects of smoking/secondhand smoke on pregnancy and children at home (33, 38). People with higher health literacy received significantly more prenatal counseling than other women and had a planned pregnancy (39).

Other studies showed that women with inadequate health literacy started prenatal care at a later gestational age, sought advice from obstetricians just before delivery, showed more symptoms of depression in the postnatal period and breastfed their babies less frequently (40).

Our research showed that women with adequate level of health literacy were more committed to chosen gynecologist, which could be related to the positive comments from their doctors.

Numerous studies that tested interventions designed to reduce differences in understanding reproductive health information, by using informative brochures on chlamydia and symptoms of cervical cancer and breast cancer—showed that women with low health literacy had significantly lower knowledge, compared to women with high health literacy after reading such brochures (40, 41).

The abovementioned conclusions also confirmed our results that women with inadequate health literacy had lack of knowledge about HIV, HPV viruses and places where they could get tested, and that the number of times they visited their doctor for regular preventive programs was much reduced.

A study examining the relationship between health literacy and quality of prenatal care found that women with lower health literacy were more likely to use the Internet as a source of information about prenatal care and smoking during pregnancy, compared to women with high health literacy (42). Women with adequate health literacy were more likely to participate in health interventions related to the literacy and advancement of reproductive health knowledge, to read more informative brochures on sexually transmitted diseases, and demonstrate a higher level of knowledge about gynecological cancer symptoms (9). Our results showed that women with adequate health literacy were 2.2 times more likely to follow the health-related themes and were 1.7 times more likely to know more about HIV and HPV, which greatly influenced the preservation of women's reproductive health behaviors.

Nowadays, ~87% of cervical cancer deaths occur due to the lack of awareness within the female populations and certain difficulties in running cervical cancer screening programs (the PAP-test). Every third woman in Serbia (35.4%) has never done a PAP-test in her lifetime. The highest percentage of respondents did their PAP-tests after they were recommended by doctors (52.3%); 45% of women did it on their own initiative, and only 2.7% did it after they had been summoned to participate in an organized screening by their doctor. The most important factors in women who had never undergone PAP-tests were the following: age (being within the youngest or the oldest age group), rural residence and low level of education, poor socio-economic status, and marital status (have never married) (43).

Research about the relationship between cervical cancer screening and health literacy revealed that women with high levels of health literacy were twice as likely to understand the purpose of PAP testing as a screening test, compared to women with inadequate health literacy. In addition, high health literacy was associated with less misunderstanding and more perceived respect by healthcare providers (34). Our findings showed great similarity in the odds of being subjected to the PAP-test among women with adequate health literacy and they were 1.7 times more likely to undergo the PAP-test.

There is no doubt that the health literacy of women can significantly affect different aspects of reproductive health such as contraception, fertility, behavior during pregnancy, childbirth and post-partum period, participation in screening examinations, knowledge of sexually transmitted diseases and sexual health. Considering the fact that there is evidence that the level of health literacy may be associated with reproductive health (44), raising the level of women's health literacy and education complete with enhancing women's legal and social status, will help women in making the right choices about their reproductive health and its promotion (45, 46). Health policy should adopt a multidimensional approach and develop incentives for the appropriate use of health services and should eliminate barriers which restrict the accessibility and availability (47).

Our study had several limitations: a small number of female respondents from specific regions of the country, then self-reporting that was prone to response biases, and factors such as affordability and accessibility that might affect the participation rate in cervical and breast cancer screenings. Further research is needed to be conducted by monitoring a larger number of different factors for more adequate identification of inequalities in women's health literacy. Future research could be conducted in other parts of the country, such as Southern and Eastern Serbia, which are considered to have different cultural and ethnic background, because there one may expect to encounter greater differences in health literacy levels, which can enable us to generate more precise data comparisons.

The importance of this study lies in the fact that this is the first study of health literacy and its relationship with reproductive health behaviors of women in Serbia based on the HLS-EU-Q47 which is used for the assessment of health literacy. These kinds of studies require special attention since women are vulnerable population groups.

It is necessary to use effective strategies for regular monitoring of the health literacy of women, as well as the implementation of prevention programs and the use of educational materials to raise the level of health literacy in order to minimize the potential negative effects of low health literacy levels on reproductive health, thus contributing to reducing inequalities in women's reproductive health and strengthening of women's involvement in the prevention and promotion of reproductive health.

## Conclusion

Based on the calculated health literacy index, significant differences were observed in health literacy levels among women in our study. Middle-aged women, women from rural areas, and those who rated their health as bad and very bad had significantly higher levels of inadequate health literacy. In relation to the surveyed characteristics of reproductive health behavior, significant differences in health literacy levels were observed among women who did not use any methods of protection against sexually transmitted diseases and unplanned pregnancies, as well as in those who did not know where to get tested for HIV, or those who did not know what damage could be caused by the HPV, or those who were not aware of HPV or hepatitis B vaccine availability. Limited health literacy and its impact on women's health are important challenges for policymakers and particularly for healthcare professionals dealing with reproductive health of the female population. Significant positive effects on improving health literacy levels in women can be achieved by increasing the availability of reproductive health protection, by promoting healthy lifestyles, by empowering women and their active participation in the community. In addition, multidisciplinary work and cooperation of the Ministry of Health with various educational institutions, sports associations, public media, local self-government, non-governmental and humanitarian organizations and associations—can significantly contribute to women's overall health and well-being.

## Data Availability Statement

The raw data supporting the conclusions of this article will be made available by the authors, without undue reservation.

## Ethics Statement

The studies involving human participants were reviewed and approved by Faculty of Medical Sciences, University of Kragujevac, Kragujevac, Serbia, 01-13458. The patients/participants provided their written informed consent to participate in this study.

## Author Contributions

MM, GS, and VP: conception of work. MS, OD, and IM: design of the work. VM, VV, SRado, and SRade: the acquisition and analysis. MM, GS, VM, VV, and SRado: interpretation of data. MM, GS, VP, and MS: drafted the work and substantively revised it. All authors: read and approved the final manuscript.

## Conflict of Interest

The authors declare that the research was conducted in the absence of any commercial or financial relationships that could be construed as a potential conflict of interest.
